# Chronic Chills in Children

**Published:** 1885-10

**Authors:** Wm. Steinrauf

**Affiliations:** Nokomis, Ill.


### CHRONIC CHILLS IN CHILDREN.

WM. STEINRAUF, M. D., NOKOMIS, ILL.

Some two months ago Mrs. John Kippen brought her two-year-old baby to my office saying: "Doctor, this boy has now had the chills for the last nine months, and, in spite of all the patent medicines, such as Smith's Tonic, Ayer's Ague Cure, etc., in spite of Quinine an Cinchonidia, the disease will not abate; in fact, the child is getting weaker every day. At times the chills occur twice a day, again once a day, at other times every second day, then once in three days." Could I "break" the chills? that is what the mother wanted to know.

Having for the last year and a half studied Homœopathy privately to some extent, I began to investigate the symptoms and to see whether or no I would be able to find the indicated remedy according to homœopathic principles, and I had no reason to regret it.

The following symptoms of the child seemed to me all to point to a certain remedy:

The type of the chills quotidian; double quotidian, tertian. Regular paroxysms—chill, fever, sweat. This indicated Sulphur. The time of the chills was not characteristic—at all periods, morning, afternoon, evening, night, chills without thirst; by heat with thirst—also Sulphur symptoms. We read in Allen's treatise on intermittent fever regarding Sulphur "that it (Sulphur) bears the same relation to chronic cases that Ipecac does to acute, viz.: if the indications for the remedy be not clear and well defined, *Sulphur may clear up the case, or completely cure it alone.* Intermittent fever is a terrible searcher after weak organs; and Sulphur is frequently required in all forms of the disease—acute and early, or chronic and later—to combat some latent malady aroused during the course of the fever. If we would use Sulphur more and Quinine less, our success would be much more satisfactory both to our patients and ourselves.

Sulphur $^{30x}$, three drops in one ounce of water, and a dose morning and evening. No more paroxysms after the first dose, and a most satisfactory recovery, was the result.

A week later, a six-year-old boy was brought to me who had now had the third-day ague for over one year. Two allopaths had tried their luck for months at a time, but to no result. The symptoms being similar to those of the first child, I administered the same remedy, only in the 3x instead of 30x, thinking that the lad needed the dose a little stronger. The first dose did not cure. Boy had three more spells, each succeeding one lighter than the one preceding.

This last case taught me a lesson, *i. e.*, to depend more on the higher attenuations. It also gave me a clue why it was I did not cure two patients last year with the third-day ague that I tried to cure with the 3x of the indicated remedy. The chills grew somewhat less, patients felt better, but they had relapses, and finally drifted into the hands of an allopath in our town, who took the cases with the understanding, "no cure, no pay."

---

ANY one having treated cases of *purpura*, which they can report in detail, showing the homœopathic applicability of any remedy, are respectfully urged to send the same to Dr. Winterburn, editor of the *American Homœopathist*, 29 West Twenty-sixth Street, New York.

---

"BACTERIA" is having a literature of its own. The latest item is *The Technology of Bacteria Investigation*, by Dr. C. S. Dolly, a well-known specialist. The volume contains directions for the study of bacteria; also hints on their culture, staining, mounting, etc.

